# Topography of small vessel cerebrovascular disease differentially impacts cognitive domains across cognitive syndromes

**DOI:** 10.18632/aging.206336

**Published:** 2025-11-17

**Authors:** Jia Dong James Wang, Yi Jin Leow, Ashwati Vipin, Gurveen Kaur Sandhu, Chao Dang, Seyed Ehsan Saffari, Nagaendran Kandiah

**Affiliations:** 1 Lee Kong Chian School of Medicine, Nanyang Technological University, Singapore 308207, Singapore; 2 Dementia Research Centre (Singapore), Lee Kong Chian School of Medicine, Nanyang Technological University, Singapore 308207, Singapore; 3 Neuroscience and Mental Health Programme, Lee Kong Chian School of Medicine, Nanyang Technological University, 308232, Singapore; 4 The First Affiliated Hospital, Sun Yat-sen University, Guangzhou 510275, China; 5 Duke-NUS Medical School, National University of Singapore, Singapore 169608, Singapore

**Keywords:** Alzheimer disease, dementia, Asian, cognition, dementia, vascular, cerebrovascular disorders

## Abstract

Introduction: Dementia in Asia, presents distinct neuropathological features, with White Matter Hyperintensities (WMH) emerging as critical indicators of small vessel disease. WMH, classified into Deep White Matter Hyperintensities (DWMH), Periventricular Hyperintensities (PVH), and Fazekas-Total, exhibit high prevalence in Asian populations. Although WMH are prevalent and recognized indicators of small vessel disease, the domain-specific cognitive impact of WMH location remains unclear. We hypothesized that examining both cognitively normal (CN) and mild cognitive impairment (MCI) groups separately would clarify whether WMH topography exerts distinct effects at different stages of cognitive decline.

Methods: 430 participants were recruited, and a cross-sectional analysis performed. Eight domains of cognition were assessed: global cognition, learning/memory, language, executive function, attention, visuo-spatial, working memory, and processing speed. Correlation and stepwise regression (with False Discovery Rate correction) were performed. 217 participants were classified as Mild Cognitive Impairment (MCI) and 213 participants as Cognitively Normal (CN) as per National Institute on Aging-Alzheimer's Association criteria.

Results: In CN participants, higher Fazekas-Total was associated with impaired attention (*p* = 0.015, β = 0.422) while higher DWMH was associated with poorer learning and memory (*p* = 0.460, β = 0.003). In MCI, higher Fazekas-Total was associated with poorer learning and memory (*p* = 0.195, β = 0.0313).

Discussion: This study demonstrates that WMH burden—particularly DWMH—exerts differential domain-specific effects in CN versus MCI populations, underscoring the importance of WMH topography in early cognitive changes. Our results suggest that evaluating DWMH separately from overall WMH may refine clinical assessments and mechanistic understanding of vascular contributions to cognitive impairment. Future research should target upstream pathophysiological processes underlying region-specific WMH to improve early detection and intervention strategies.

## INTRODUCTION

Dementia is a growing global health concern, currently affecting 55 million people, with projections reaching 152.8 million by 2050 [1–3]. Asia bears 60% of this burden, driven by ageing populations, underscoring the need for deeper insights into the disease’s biology, progression, and clinical manifestations. In this region, Alzheimer’s disease and vascular dementia are the most prevalent forms [4], presenting complex challenges for public health and clinical care. Targeted research is essential to refine diagnosis, treatment, and prognosis, particularly in Asia, where the impact of dementia is rising rapidly due to disproportionately high burden of ageing populations.

White Matter Hyperintensities (WMH), which are white matter lesions visualized on Magnetic Resonance Imaging (MRI), are key markers of small vessel disease (SVD) [5]. SVD is a pathological process where vessel walls in small vessels like capillaries and arterioles are damaged, leading to decreased perfusion to the brain. SVD is prevalent in Asian populations and is strongly associated with both prodromal and clinical stages of dementia [4]. Current studies have shown a relatively high prevalence of SVD and therefore WMH in the Asian population, with some studies showing up to 36.6% of participants having WMH [6].

WMH is traditionally described as: Deep White Matter Hyperintensities (DWMH), Periventricular Hyperintensities (PVH) or Fazekas Total (DWMH and PVH) [7, 8]. Current studies have shown that higher WMH burden among patients were associated with poorer performance in global cognition, memory, and executive function [9]. In addition, a recent paper found that executive function, memory and visual memory were associated with WMH in participants with subjective cognitive decline (SCD). However, the association between WMH topography and performance in specific cognitive domains like social cognition remains unclear [10]. Specifically, there has been few studies quantifying the specific type of WMH (PVH or DWMH) and their individual impact on different domains of cognition, with the majority quantifying WMH as a combination of PVH and DWMH: Fazekas Total [9]. Therefore, we wish to investigate this in a Southeast Asian population, across broader groups comprising of cognitively normal to cognitively impaired.

Building on this knowledge gap, we aim to explore the differential impacts of Fazekas Total, DWMH, and PVH on specific cognitive domains—including global cognition, learning and memory, language, executive function, attention, working memory, visuospatial skills, and processing speed—in a Southeast Asian prodromal dementia cohort. By examining both cognitively normal and cognitively impaired individuals, this study will provide a more nuanced view of how WMH topography influences cognition at varying stages of disease progression. Our findings could advance clinical decision-making for dementia risk assessment, diagnosis, and prognosis in Southeast Asia, thereby informing early intervention and potentially mitigating disease burden.

## METHODS

### Study design

We conducted a cross-sectional analysis using data from 430 participants enrolled in the Biomarkers and Cognition Study, Singapore BIOCIS. BIOCIS aims to characterize biomarker profiles, neuroimaging features, and neuropsychological and clinical outcomes within a multi-ethnic Southeast Asian cohort. Participants were recruited from community settings in Singapore between February 2022 and July 2023. For comprehensive details on the study’s design, methodology, and procedures, please refer to the published BIOCIS Protocol [11].

### Participant recruitment

The study included participants with intact mental capacity and proficiency in either English or Mandarin. Eligibility was restricted to individuals classified as cognitively normal (CN) or diagnosed with mild cognitive impairment (MCI), provided they had undergone comprehensive neuropsychological assessments and MRI scans. Exclusion criteria encompassed a history of psychotic disorders, clinically significant neurological conditions such as stroke, severe systemic illnesses, alcoholism, or drug dependence within the past two years, as well as individuals with subjective cognitive decline (SCD).

The participants were classified as CN or MCI as per the National Institute on Aging-Alzheimer’s Association (NIA-AA) criteria [12] and published literature [13].

Participants with a Clinical Dementia Rating (CDR) of 0, no subjective cognitive complaints, and a Montreal Cognitive Assessment (MoCA) score >26 were classified as cognitively normal (CN). Participants who reported cognitive symptoms on the Subjective Memory Complaints Questionnaire (SMCQ) without functional deficits but showing objective cognitive impairments (performance >1.5 SD below the mean on cognitive tests), were classified as MCI. Participants with a CDR >1 or who exhibited functional deficits were classified as demented and excluded from the study.

### Neuropsychological and demographics assessment protocol

Cognition was assessed by trained raters utilizing standardized neuropsychological assessments which includes CDR Scale [14], Montreal Cognitive Assessment (MoCA) [15], Visual Cognitive Assessment Tool (VCAT) [16], Rey Auditory Verbal Learning Test (RAVLT) [17, 18], Rey–Osterrieth Complex Figure (ROCF) [19], Free and Cued Selective Reminding Test (FCSRT) [20], Test of Practical Judgment (TOP-J) [21], Colour Trails Test A (CTT-A) [22], Colour Trails Test B (CTT-B) [22], Trail Making Test B (TMT-B) [23], Semantic fluency [24], WAIS Digit Span Backwards [25], WAIS Digit Span Forward [26], WAIS Block Design Test [26] and Symbol Digit Modalities Test [27]. The test spanned across eight domains of cognition, which is shown in the Supplementary Figure 1.

Demographics such as age, gender and years of education were self-reported by participants. Additionally, a self-reported standardized lifestyle and behavioral questionnaires - Subjective Memory Complaints Questionnaire (SMCQ) was included [28].

### Magnetic resonance imaging (MRI) protocol

The participants underwent neuroimaging assessments using a 3T Siemens Prisma Fit (Siemens, Erlangen, Germany) MRI machine. The T1-weighted MPRAGE sequence was acquired with repetition time of 2000 ms, echo time 2.26 ms, inversion time 800 ms, flip angle 8, 1 mm slice thickness, 176 number of slices, and 1 × 1 × 1 mm voxel size. The T2-weighted FLAIR MRI sequence was acquired with repetition time of 7000 ms, echo time 394 ms, inversion time 2100 ms, flip angle 120, 1.56 mm slice thickness, 192 slices, 0.8 × 0.8 × 1 mm voxel size.

#### Rating for WMH features pf PVH, DWMH and fazekas total

To quantify the WMH features of PVH, DWMH and Fazekas Total, MRI visual ratings were performed on T1 and FLAIR MRI sequences by two trained raters blinded to diagnosis according to previously published methods [8] as shown in Supplementary Figure 2. DWMH were differentiated when lesions are more than 10 mm away from the lateral ventricles, in line of previous published literature [29].

For PVH, it was rated as “0” if hyperintensities were absent, “1” if hyperintensities showed “caps” or pencil-thin lining (<5 mm), “2” if hyperintensities showed smooth “halo” (5 mm to <10 mm) and “3” if hyperintensities showed irregular periventricular signal extending into the deep white matter (≥10 mm). For DWMH, it was rated as “0” if hyperintensities were absent, “1” if hyperintensities showed punctate foci (<5 mm), “2” if hyperintensities showed beginning confluence (5 mm to <10 mm) and “3” if hyperintensities showed large confluent areas (≥10 mm) [8].

For PVH and DWMH, they were graded on a scale of 0–3 [30] for each hemisphere of the brain (right versus left), giving a total score ranging from 0–6 after summation of right and left scoring for the total PVH or DWMH scoring for each participant. The Fazekas Total score was calculated through summation of PVH and DWMH scoring, giving a total score ranging from 0–12.

### Statistical analysis

Statistical analysis of results was performed using R 4.2.2 version, Python 3.11 and IBM SPSS Statistics 26. Statistical significance was set as *p* < 0.05. Normality was tested using Q-Q plots [31] and Shapiro-Wilk (SW) tests [32] for the WMH features (PVH, DWMH and Fazekas Total) and neuropsychological assessments Supplementary Tables 1 and 2. Any timed tests had absolute value transformation for simplicity of interpretation.

#### Mapping of cognitive domains affected in CN and MCI groups

Domain-level z-scores were calculated by taking the mean of standardized (z-transformed) raw test scores within each domain and outlined in a heatmap. This was conducted to compare the domains of cognition affected by WMH in the subsequent analysis. For tests that contribute to multiple domains (e.g., Colour Trails B for executive function and processing speed), the primary domain assignment was used in domain averaging, with secondary contributions noted in supplementary materials. We chose this mean-of-z approach to facilitate interpretability in heatmaps comparing group-level domain profiles.

#### Association between Neuropsychological test scores and WMH features

Correlation testing was carried out between each WMH feature and neuropsychological assessment. If the data for both the specific WMH feature and neuropsychological assessment were normally distributed, a Pearson’s correlation was utilised [33]. If either of them were not normally distributed, a Spearman’s correlation was utilised [33]. For neuropsychological assessments which were time-based like CTT-A, an absolute value correction was used for the correlation coefficients to enable comparability between different neuropsychological assessments. For inferential analyses (correlations and regressions), individual raw test scores were used rather than domain-level z-scores. This decision preserves test-level variability and statistical power while allowing adjustment for covariates. We clarify that domain z-scores were used descriptively (for heatmap visualization of average impairment), whereas raw scores were modeled for hypothesis testing of WMH-cognition associations.

#### Stepwise regression of cognitive tests and WMH features

A forward stepwise regression [34] was run for PVH, DWMH and Fazekas Total against every neuropsychological assessment that had significant correlation (*p* < 0.05) in the previous correlation analysis. Previous neuropsychological assessments which showed negative correlation were corrected for comparison purposes (through negation of each assessment value).

This was to understand the relative contributions of each WMH feature (for PVH, DWMH and Fazekas Total) to impairment in the eight domains of cognition while controlling for age, gender and education years. The independent variables were WMH features (PVH, DWMH and Fazekas Total) and the dependent variables were the specific neuropsychological assessments.

Multiple-comparison correction was carried out using the Benjamini and Hochberg False Discovery Rate method [35] for all forward stepwise regression. β-values were analyzed to understand the degree of impairment of different domains of cognition contributed by each type of WMH. We chose stepwise regression as it offers a pragmatic approach to identify the most relevant WMH features contributing to cognitive domain performance while adjusting for demographic covariates like age, gender and education years. This approach is particularly useful in settings with multiple, potentially correlated predictors.

### Data availability statement

Data are not publicly available and may be made available by the corresponding author upon reasonable request.

## RESULTS

Overall, 430 participants were included in the final analysis. 217 participants were classified as MCI and 213 participants as CN. The mean, standard deviation and range of demographics (including gender, age, education years), WMH features (Fazekas Total, PVH and DWMH) and global cognitive tests (MoCA, VCAT) were summarized in Table 1. There were significant differences associated with the different subgroups of MCI and CN (*p* < 0.05) in the demographics, neuroimaging, and cognitive test results Supplementary Table 3.

**Table 1:** Demographics, neuroimaging features, and cognitive test scores of the study population.

		**CN participants *n* = 213**	**MCI participants *n* = 217**	***p*-value**
**Demographics**				
**Age (years)**		57.3 ± 11.1	64.4 ± 9.09	<0.001
**Gender (Male)**		109 (50.2%)	90 (42.3%)	0.013
**Years of education**		15.0 ± 3.35	13.35 ± 4.13	<0.001
**Neuroimaging Features**				
**Fazekas Total**		3.66 ± 2.71	4.59 ± 2.91	0.002
**Periventricular white matter**		1.4 ± 1.38	1.83 ± 1.48	0.006
**Deep white matter hyperintensities**		2.25 ± 1.65	2.76 ± 1.73	0.005
**Domain of cognition**				
**Global**	MoCA	27.7 ± 1.33	24.3 ± 2.86	<0.001
	VCAT	27.1 ± 2.48	25.3 ± 3.42	<0.001
**Learning and memory**	Rey Auditory Verbal Learning Test (RAVLT) Delayed	9.08 ± 3.50	11.87 ± 2.75	<0.001
	Rey–Osterrieth Complex Figure (ROCF) Delayed	17.88 ± 7.95	22.36 ± 6.87	<0.001
	Free and Cued Selective Reminding Test (FCSRT) Learning Delayed Free Recall	11.54 ± 2.78	13.29 ± 2.00	<0.001
**Language**	Semantic fluency	16.81 ± 4.42	20.11 ± 2.97	<0.001
**Executive function**	Test of Practical Judgment (TOP-J) B	15.59 ± 4.57	17.50 ± 4.17	<0.001
	Trail Making Test B	86.60 ± 52.81	63.75 ± 31.29	<0.001
	Colour Trails B	106.36 ± 43.60	83.93 ± 30.00	<0.001
**Attention**	WAIS Digit span Forward	6.81 ± 1.33	7.25 ± 1.47	0.006
**Working memory**	WAIS Digit span Backwards	4.78 ± 1.52	5.71 ± 1.60	<0.001
**Visuospatial**	WAIS Block design	34.73 ± 8.22	38.32 ± 7.38	<0.001
**Processing speed**	Symbol Digit Modalities Test	62.92 ± 18.02	75.38 ± 15.68	<0.001
	Colour Trails A	52.94 ± 20.88	43.32 ± 17.66	<0.001

Abbreviations: CN: Cognitively Normal; MCI: Mild Cognitive Impairment; MoCA: Montreal Cognitive Assessment; VCAT: Visual Cognitive Assessment Test. Data are presented as mean ± standard deviation unless otherwise stated. Bold values denote statistical significance at *p* < 0.05.

### Cognitive domains affected in CN, SCD and MCI groups

When comparing average z-scores, visuospatial, executive function and working memory (−0.070) domain tests had the lowest average z-score in CN participants. In MCI participants, executive function (−1.11) domain tests had the lowest average z-score as shown in Table 2.

**Table 2:** Average z-scores of various cognitive tests in between groups of CN and MCI.

**Domains**	**Cognitive test**	**CN (Mean ± SD)**	**MCI (Mean ± SD)**
**Learning and memory**	Rey Auditory Verbal Learning Test	0.10 ± 0.93	−0.84 ± 1.21
	Free and Cued Selective Reminding Test (FCSRT) Learning	0.29 ± 0.77	−0.42 ± 1.16
	Rey–Osterrieth Complex Figure	−0.02 ± 1.01	−0.66 ± 1.18
**Language**	Semantic fluency	0.16 ± 0.85	−0.73 ± 1.22
**Executive function**	Trail Making Test B	−0.07 ± 1.39	−1.11 ± 2.38
	Colour Trails B	−0.07 ± 1.37	−1.02 ± 1.92
	Test of Practical Judgment B	0.21 ± 0.90	−0.20 ± 0.99
**Working memory**	WAIS Digit Span Backwards	−0.07 ± 0.95	−0.73 ± 0.91
**Attention**	WAIS Digit Span Forward	0.14 ± 1.01	−0.18 ± 0.97
**Processing speed**	Colour Trails A	0.10 ± 1.06	−0.47 ± 1.14
	Symbol Digit Modalities Test	0.04 ± 0.95	−0.67 ± 0.90
**Visuospatial**	WAIS Block Design	−0.07 ± 1.06	−0.52 ± 1.00

Average z-scores are shown within each group (CN and MCI). Abbreviations: CN: Cognitively Normal; MCI: Mild Cognitive Impairment.

### Correlation analysis of cognitive tests and WMH features

#### WMH features correlation with neuropsychological assessments (CNMCI participants)

Fazekas Total, PVH and DWMH had strong correlation to processing speed in CN participants (Rho = 0.294, 0.225, 0.294) as shown the supplementary material. Fazekas Total, PVH and DWMH had strong correlation to processing Speed in MCI participants (Rho = 0.278, 0.223, 0.281) as seen in the Supplementary Table 4.

### Stepwise regression of cognitive tests and MRI features

Table 3 details the association between specific White Matter Hyperintensities features (Deep White Matter Hyperintensities, Periventricular Hyperintensities and Fazekas Total) and different domains of cognition, with the neuropsychological test utilized outlined. Only three domains of cognition performance tested were influenced by WMH and results were demonstrated in Table 3.

**Table 3:** A summary of stepwise regression results for cognitively normal and mild cognitive impairment participants.

**Domains assessed**	**Neuropsychological assessment**	**Fazekas total**		**PVH**		**DWMH**	
		**β-value**	**Adjusted *p*-value**	**β-value**	**Adjusted *p*-value**	**β-value**	**Adjusted *p*-value**
**Learning and memory**	RAVLT	0.210 (−0.011–0.410)	0.064	0.195 (−0.029–0.361)	0.052	0.460 (0.215–0.704)	0.003
**Executive function**	Trail Making Test	0.014 (−0.0011–0.0264)	0.064	NS	NS	NS	NS
**Attention**	WAIS Digit span Forward	0.422 (0.133–0.710)	0.015	0.13 (−0.004–0.253)	0.052	0.038 (−0.005–0.071)	0.062
**Learning and memory**	ROCF Immediate	0.195 (0.018–0.372)	0.0313	0.004 (−0.0006–0.010)	0.114	0.0759 (−0.279–0.180)	0.151
**Executive function**	Colour Trails B	0.08 (0.01–0.179)	0.0106	NS	NS	NS	NS
**Attention**	−	NS	NS	NS	NS	NS	NS

For the domains of cognition that were overlapped in each of the neuropsychological assessment, the dominant domain was indicated via. Abbreviations: RAVLT: Rey Auditory Verbal Learning Test (RAVLT); ROCF Immediate: Rey–Osterrieth Complex Figure; NS: Non-significant.

For CN participants, Fazekas Total mainly affected attention (*p* = 0.015, β = 0.422). For DWMH, the greatest impact was on learning and memory (*p* = 0.003, β = 0.460) impairment. For MCI participants, Fazekas Total was significantly associated with learning and memory (*p* = 0.0313, β = 0.195) impairment, followed by executive function impairment (*p* = 0.0106, β = 0.08).

**Figure 1:**
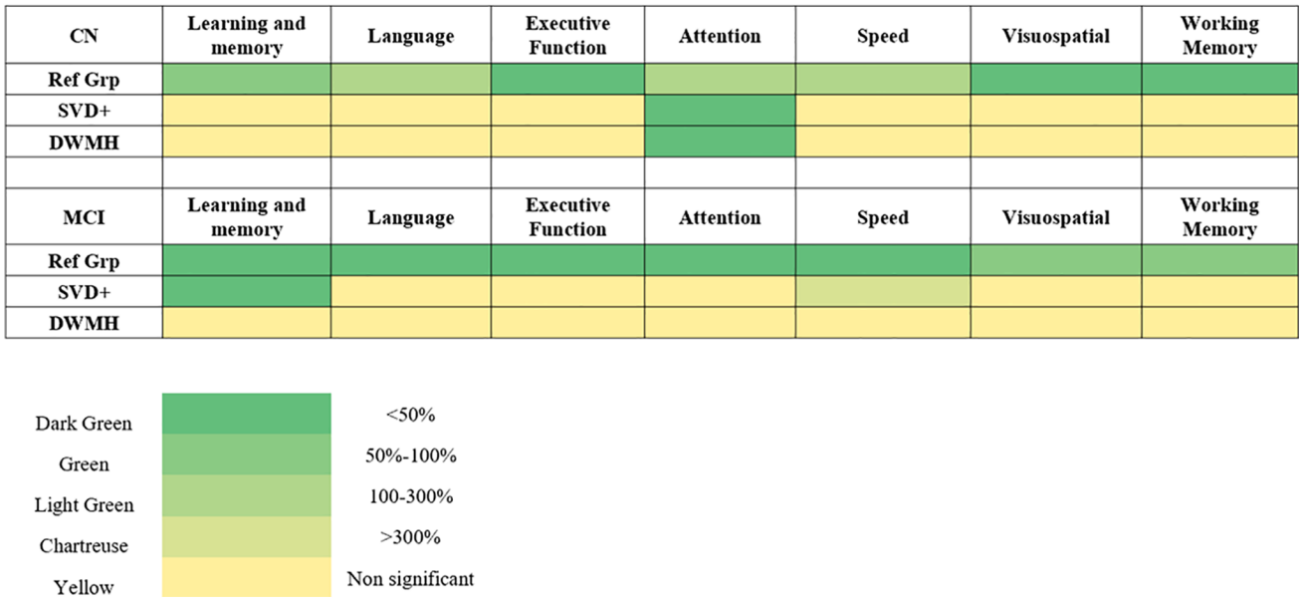
**A heatmap illustrating the relative impairment of various cognitive domains across different participant groups, including cognitively normal (CN) and mild cognitive impairment (MCI).** Comparisons are made across the general phenotype (Ref Grp), participants with white matter hyperintensities (WMH; SVD+ participants), and deep white matter hyperintensities (DWMH). The heatmap uses five distinct colours to depict the severity of impairment in each domain. Dark green represents the domain with the greatest impairment (i.e., the most affected domain in each group), while progressively lighter shades indicate less impairment relative to this maximum. Specifically, green indicates 50–100% of the maximal impairment, light green corresponds to 100–300%, chartreuse represents >300%, and yellow indicates non-significant differences.: dark green represents the domain with the greatest impairment, including any domain showing less than 50% of this impairment relative to the most affected domain. Green indicates impairments between 50–100%, light green corresponds to impairments from 100–300%, and chartreuse represents impairments exceeding 300%. Yellow represents domains that are non-significant. Abbreviations: CN: cognitively normal; MCI: mild cognitive impairment; WMH: white matter hyperintensities; SVD: small vessel disease.

A heatmap was created (Figure 1) to showcase the relative differences in domain of cognition affected in the general populations and contrasting it to when white matter hyperintensities or SVDs are accounted for.

## DISCUSSION

Our study explored the relationship between WMH and various cognitive domains across participants with different stages of cognitive impairment—CN and MCI. The findings highlight significant associations between specific WMH features (Fazekas Total, PVH, DWMH) and cognitive function, demonstrating that different types of WMH influence distinct cognitive domains.

### WMH and cognitive domains

The results indicate that WMH features, particularly DWMH, may differentially impact cognitive domains. DWMH was most strongly associated with learning and memory impairments, as observed in CN (β = 0.46). The significant impact of DWMH on learning and memory suggests that WMH distribution patterns may hold greater clinical relevance than total burden, though causality cannot be established in this cross-sectional design. This is consistent with prior research, which suggests that white matter tracts, such as the superior longitudinal fasciculus and uncinate fasciculus, could be involved in learning and memory processes [36]. The diffused nature of DWMH may lead to more widespread cognitive disruptions, particularly in memory-related functions [37]. While we did not include tractography in this study, we propose this as a mechanistic hypothesis for future work. This could be clinically significant, as MRI can be used to individually characterize the burden of DWMH, allowing for tailored treatment plans.

### Specific WMH features in MCI, SCD, and CN

In participants with MCI, learning and memory impairment showed the strongest association with Fazekas Total WMH burden (β = 0.195). This finding is consistent with literature indicating that memory deficits are central to MCI [38]. Furthermore, the strong relationship between WMH burden and learning and memory suggests that WMH could serve as a key imaging marker for identifying individuals at higher risk of progressing from MCI to Alzheimer’s disease.

In CN participants, Fazekas Total was significantly associated with complex attention impairments (β = 0.422). This suggests that attention deficits may emerge early in the dementia continuum, possibly reflecting the early stages of cognitive decline related to SVD. Attention impairment, as part of the attention-encoding-storage-retrieval framework, may be an initial manifestation of neurodegeneration, particularly in populations at high risk for dementia [39].

### Clinical implications

A comparison of Figure 1 reveals that cognitive domains were differentially affected in participants with and without WMH burden. For example, in MCI participants, executive function, working memory and visuospatial memory was most impaired when WMH was not considered, but when WMH burden was factored in, learning and memory emerged as the most affected domain. This finding aligns with existing literature suggesting that MCI in Southeast Asia may present with greater executive function impairments, but that WMH burden shifts the cognitive impact toward memory deficits.

These findings have important clinical implications. The differential impact of WMH on specific cognitive domains highlights the need for more nuanced clinical phenotyping as shown in Figure 1. Assessing cognitive function with consideration of WMH burden allows for a more precise diagnosis and prognosis, as different cognitive domains may be influenced by WMH in various stages of cognitive decline. This targeted approach could guide more personalized interventions for patients at different stages of dementia, particularly in Southeast Asian populations. Moreover, clinicians could consider incorporating structured WMH assessments—separately rating DWMH and PVH—into routine MRI protocols for patients with suspected early cognitive impairment. Such neuroimaging-informed evaluations may support more precise risk stratification, facilitate early diagnosis, and inform targeted cognitive interventions tailored to domain-specific deficits. However, we did not define practical MRI thresholds for DWMH severity to guide such interventions, and current evidence for using WMH imaging to personalize cognitive stimulation therapy remains limited; future studies should assess feasibility and validate thresholds prospectively.

### Strengths and limitations

A key strength of this study is its population-based design, drawing participants from a community cohort in Singapore. This enhances the generalizability of our findings to real-world settings, as opposed to hospital-based samples. Moreover, analyzing multiple cognitive domains illuminates the differential effects of WMH features. Importantly, this is the first systematic analysis of the association between WMH subtypes (DWMH, PVH) and specific cognitive domains in a Southeast Asian population, directly addressing a regional research gap.

Nevertheless, certain limitations must be acknowledged. First, our cross-sectional design limits causal inferences (as WMH can co-occur with neurodegeneration), although a five-year longitudinal follow-up is underway to clarify temporal relationships and disease progression. We also acknowledge that MRI resolution differences (1.5T vs. 3T) could influence WMH detection and comparability. Moreover, we recognize that although WMH–cognition associations are well-studied, our contribution focuses on subtype-specific patterns in a Southeast Asian context rather than introducing new mechanistic biomarkers. Second, while this study centers on global WMH burden, future research would benefit from evaluating regional WMH patterns to pinpoint topographic effects on specific cognitive domains. Additionally, the study did not control other variables that affect WMH such as vascular risk factors like hypertension, diabetes or lifestyle factors like exercise as the data was not collected, limiting casual interpretation of results. APOE4 status was also not recorded, therefore could not be analyzed. Another limitation of our study is the reliance on consensus-based Fazekas visual ratings without formal reporting of inter-rater reliability (e.g., κ or ICC), which may introduce subjective variability. Additionally, Fazekas scores provide limited regional detail, potentially obscuring nuanced topographic effects of WMH on cognition. Future work should include reliability metrics and consider semi-quantitative volumetric analyses to improve precision. Furthermore, there was insufficient adjustment for education level beyond inclusion as a covariate, despite high educational heterogeneity in Southeast Asian populations, which may confound cognitive test performance. Lastly, there is potential variable selection bias due to the stepwise regression model utilized.

### Future directions

Future research should aim to compare the BIOCIS cohort longitudinally with international cohorts such as the Alzheimer’s Disease Neuroimaging Initiative (ADNI) to evaluate the consistency of WMH-cognition associations across diverse populations. Additionally, examining the impact of specific WMH locations, particularly along key white matter tracts like the superior longitudinal fasciculus, could provide deeper insights into how lesion topography influences cognitive domains, shedding light on the underlying pathophysiology of dementia. Further investigation is also warranted to assess the role of perivascular spaces and their contributions to various cognitive domains, particularly within Southeast Asian populations [40]. Additionally, future studies should investigate upstream pathophysiological mechanisms underlying region-specific WMH development, such as blood–brain barrier dysfunction, chronic cerebral hypoperfusion, endothelial injury, and neuroinflammation. Clarifying these mechanisms could enable biomarker discovery and guide targeted prevention strategies for small vessel disease–related cognitive decline. Finally, while this study focuses on a Southeast Asian cohort, the generalizability of these findings to non-Asian populations remains to be determined, given potential differences in small vessel disease prevalence, vascular risk profiles, and genetic factors.

## CONCLUSION

Our findings demonstrate that WMH burden differentially influences cognition depending on both WMH subtype and stage of cognitive decline. Specifically, DWMH is closely linked with learning and memory impairment, whereas overall WMH burden (Fazekas Total) appears to more strongly affect attention in CN individuals and learning/memory in MCI. This nuanced view of WMH subtypes underscores the importance of precise neuroimaging assessments in early-stage dementia care. Such targeted evaluations could enable more personalized interventions and more accurate prognoses for populations at high risk of vascular-related cognitive impairment, particularly in Southeast Asia.

## Supplementary Materials

Supplementary Figures

Supplementary Tables
