# Supplementary material for: Topography of small vessel cerebrovascular disease differentially impacts cognitive domains across cognitive syndromes
Source: Aging (Albany NY). 2025 Nov 17;17(11):2744–58. doi: 10.18632/aging.206336 (PMC12705179; doi:10.18632/aging.206336)
Supplement: Supplementary Figures [file aging-17-11-206336-s001.pdf]

SUPPLEMENTARY FIGURES

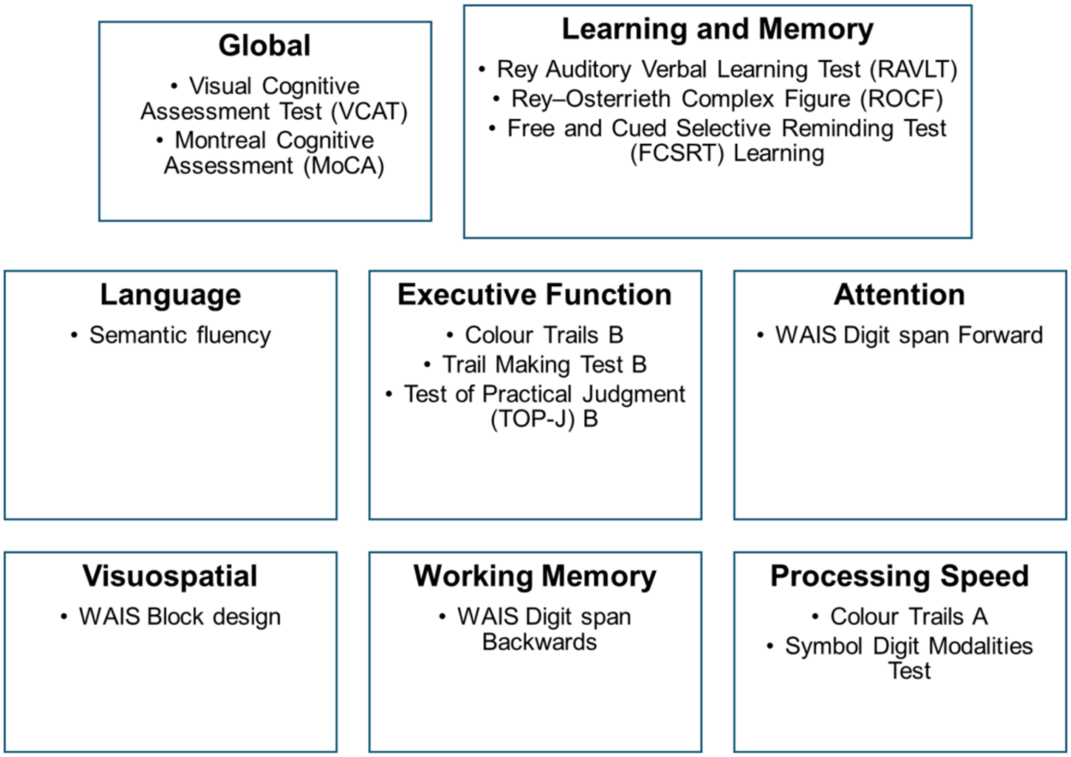

Supplementary Figure 1. List of neuropsychological assessments (cognitive tests) and respective domains of cognition assessed. When a cognitive test assesses multiple domains, the primary domain is displayed in regular text, while secondary domains are italicized and presented in grey.

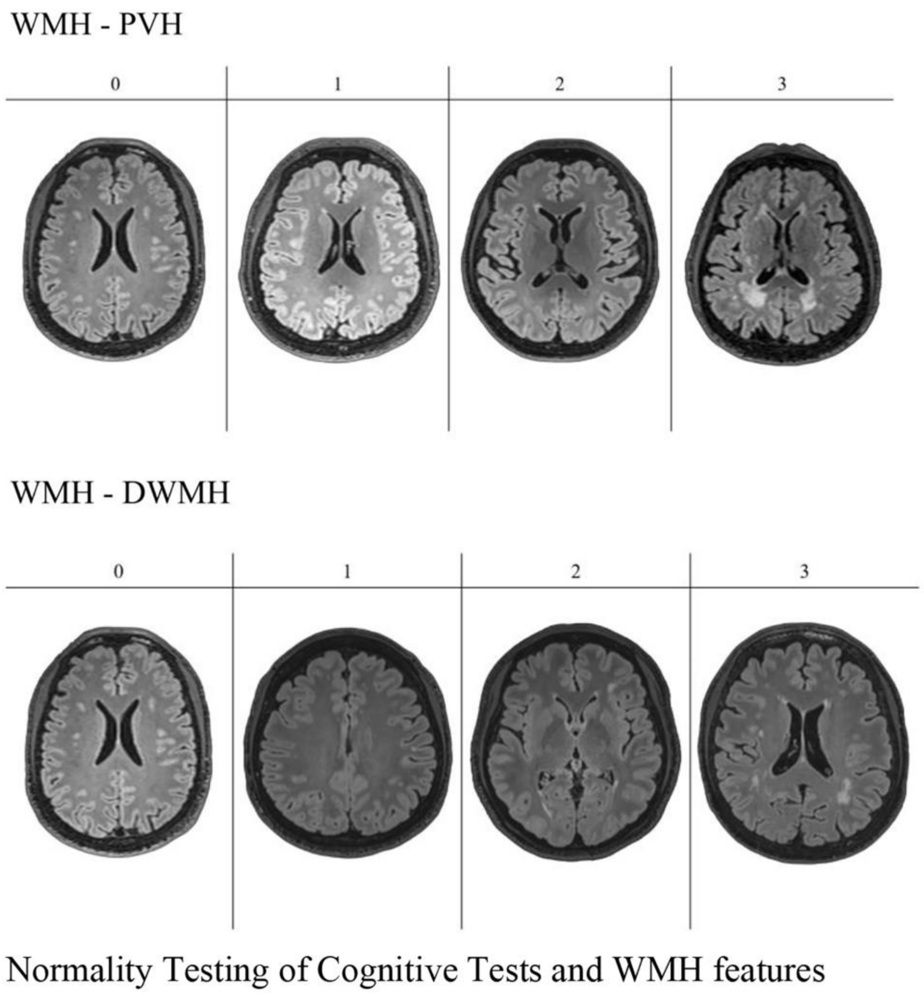

Supplementary Figure 2. Illustration of PVH and DWMH severity and rating in MRI images.
