# Supplementary material for: Topography of small vessel cerebrovascular disease differentially impacts cognitive domains across cognitive syndromes
Source: Aging (Albany NY). 2025 Nov 17;17(11):2744–58. doi: 10.18632/aging.206336 (PMC12705179; doi:10.18632/aging.206336)
Supplement: Supplementary Tables [file aging-17-11-206336-s002.pdf]

SUPPLEMENTARY TABLES

Supplementary Table 1. A summary of the Shapiro-Wilk tests results for each WMH feature (PVH, DWMH and Fazekas Total) in CN and MCI participants.

SW test *p*-value	PVH	DWMH	Fazekas Total
**CN**	1.33E-15	3.99E-10	1.71E-09
**MCI**	4.84E-14	1.05E-07	1.22E-06

As all the WMH features data distribution were non-normal as shown above, a Spearman correlation was utilised for the subsequent analysis. Abbreviations: CN: Cognitively Normal; MCI: Mild Cognitive Impairment.

Supplementary Table 2. A summary of the frequency of normal vs. non-normal Shapiro-Wilk tests results for each neuropsychological assessment.

SW test frequency table		Number of neuropsychological assessments
**CN**	$P < 0.05$	14
	$P > 0.05$	2
**MCI**	$P < 0.05$	15
	$P > 0.05$	1

Normality was tested for the rest of the neuropsychological assessments with a summary shown above, with the majority of the assessments showing non-normal distributions. A correlation analysis was done which is shown in Table 3. Abbreviations: CN: Cognitively Normal; MCI: Mild Cognitive Impairment.

Supplementary Table 3. Summary of amnestic vs. non-amnestic MCI.

SW test frequency table		Number of participants
**MCI**	Amnestic	73 (33.6%)
	Non-amnestic	144 (66.4%)

The number of amnestic MCI participants is lower than the current literature, likely because BIOCIS is a community-dwelling population.

Supplementary Table 4. A summary of correlations between specific White Matter Hyperintensities features and different neuropsychological assessment by cognitive group.

Domains assessed	Neuropsychological assessment	Cognitively normal			Mild cognitive impairment		
		Fazekas total	PVH	DWMH	Fazekas Total	PVH	DWMH
		Spearman Rho adjusted	Spearman Rho adjusted	Spearman Rho adjusted	Spearman Rho adjusted	Spearman Rho adjusted	Spearman Rho adjusted
**Global**	VCAT	0.155	NS	0.179	0.215	0.232	0.183
**Global**	MoCA	NS	NS	NS	0.265	0.164	0.279
**Learning and memory**	ROCF Immediate	0.236	NS	0.274	NS	0.146	NS
**Learning and memory**	FCSRT Learning	0.183	NS	0.212	0.172	0.225	NS
**Learning and memory**	RAVLT	NS	NS	0.141	0.165	NS	0.162
**Processing speed**	Colour Trails A	0.214	0.148	0.237	0.152	0.187	NS

**Processing speed**	Symbol Digit Modalities Test	0.294	0.225	0.294	0.278	0.223	0.281
**Executive function**	Colour Trail B	0.241	0.179	0.252	0.200	0.236	0.147
**Executive function#, processing speed**	Trial Making Test B	0.250	0.173	0.261	0.164	0.178	0.135
**Attention**	WAIS Digit span Forward	0.193	0.144	0.198	NS	NS	NS
**Working memory**	WAIS Digit span Backwards	0.169	0.171	0.147	0.191	NS	0.191
**Language**	Semantic fluency	0.220	0.150	0.220	NS	NS	NS
**Visuospatial**	WAIS Block design	0.242	0.165	0.255	0.174	0.214	NS
**Executive function**	TOP-J B	NS	NS	NS	NS	NS	NS

Only correlations where $p < 0.05$ were included. For tests that assessed various domains of cognition, the dominant domain was indicated by '#'. All reported values were corrected Rho values ($p < 0.05$) through modulation. Abbreviations: VCAT: Visual Cognitive Assessment Test; MoCA: Montral Cognitive Assessment; ROCF Immediate: Rey–Osterrieth Complex Figure; FCSRT Learning: Free and Cued Selective Reminding Test (FCSRT) Learning; RAVLT: Rey Auditory Verbal Learning Test (RAVLT); WMS-IV Logical Memory: Wechsler Memory Scale (WMS)-IV Logical Memory; TOP-J: Test of Practical Judgement; NS: Non-significant.
